# *ABCB1* Gene Polymorphisms and Their Contribution to Cognitive Decline in Mild Cognitive Impairment: A Next-Generation Sequencing Study

**DOI:** 10.1093/gerona/glaf055

**Published:** 2025-04-01

**Authors:** Omar Šerý, Kateřina Sheardová, Radka Dziedzinska, Tomáš Zeman, Martin Vyhnálek, Hana Marková, Jan Laczó, Jan Lochman, Kamila Vrzalová, Vladimir J Balcar, Jakub Hort

**Affiliations:** Laboratory of Neurobiology and Molecular Psychiatry, Department of Biochemistry, Faculty of Science, Masaryk University, Brno, Czech Republic; Laboratory of Neurobiology and Pathological Physiology, Institute of Animal Physiology and Genetics, Czech Academy of Sciences, Brno, Czech Republic; International Clinical Research Center, St. Anne’s University Hospital Brno, Brno, Czech Republic; First Neurology Department, St. Anne’s University Hospital Brno, Brno, Czech Republic; Laboratory of Neurobiology and Molecular Psychiatry, Department of Biochemistry, Faculty of Science, Masaryk University, Brno, Czech Republic; Laboratory of Neurobiology and Pathological Physiology, Institute of Animal Physiology and Genetics, Czech Academy of Sciences, Brno, Czech Republic; Laboratory of Neurobiology and Molecular Psychiatry, Department of Biochemistry, Faculty of Science, Masaryk University, Brno, Czech Republic; Laboratory of Neurobiology and Pathological Physiology, Institute of Animal Physiology and Genetics, Czech Academy of Sciences, Brno, Czech Republic; International Clinical Research Center, St. Anne’s University Hospital Brno, Brno, Czech Republic; Memory Clinic, Department of Neurology, Charles University, Second Faculty of Medicine and Motol University Hospital, Prague, Czech Republic; International Clinical Research Center, St. Anne’s University Hospital Brno, Brno, Czech Republic; Memory Clinic, Department of Neurology, Charles University, Second Faculty of Medicine and Motol University Hospital, Prague, Czech Republic; International Clinical Research Center, St. Anne’s University Hospital Brno, Brno, Czech Republic; Memory Clinic, Department of Neurology, Charles University, Second Faculty of Medicine and Motol University Hospital, Prague, Czech Republic; Laboratory of Neurobiology and Molecular Psychiatry, Department of Biochemistry, Faculty of Science, Masaryk University, Brno, Czech Republic; Laboratory of Neurobiology and Pathological Physiology, Institute of Animal Physiology and Genetics, Czech Academy of Sciences, Brno, Czech Republic; Laboratory of Neurobiology and Molecular Psychiatry, Department of Biochemistry, Faculty of Science, Masaryk University, Brno, Czech Republic; Laboratory of Neurobiology and Pathological Physiology, Institute of Animal Physiology and Genetics, Czech Academy of Sciences, Brno, Czech Republic; Neuroscience Theme, School of Medical Sciences, Faculty of Medicine and Health, The University of Sydney, Sydney, Australia; International Clinical Research Center, St. Anne’s University Hospital Brno, Brno, Czech Republic; Memory Clinic, Department of Neurology, Charles University, Second Faculty of Medicine and Motol University Hospital, Prague, Czech Republic; (Biological Sciences Section)

**Keywords:** Alzheimer’s disease, ATP-dependent translocase, ATP-binding cassette transporters, DNA polymorphisms, Language decline

## Abstract

The *ABCB1* gene, encoding the ATP-dependent translocase ABCB1, plays a crucial role in the clearance of amyloid-beta (Aβ) peptides and the transport of cholesterol, implicating it in the pathogenesis of Alzheimer’s disease. The study aims to investigate the association between polymorphisms in the *ABCB1* gene and cognitive decline in individuals with mild cognitive impairment (MCI), particularly focusing on language function. A longitudinal cohort study involving 1 005 participants from the Czech Brain Aging Study was conducted. Participants included individuals with Alzheimer’s disease, amnestic MCI, non-amnestic MCI, subjective cognitive decline, and healthy controls. Next-generation sequencing was utilized to analyze the entire *ABCB1* gene. Cognitive performance was assessed using a comprehensive battery of neuropsychological tests, including the Boston Naming Test and the semantic verbal fluency test. Ten *ABCB1* polymorphisms (rs55912869, rs56243536, rs10225473, rs10274587, rs2235040, rs12720067, rs12334183, rs10260862, rs201620488, and rs28718458) were significantly associated with cognitive performance, particularly in language decline among amnestic MCI patients. In silico analyses revealed that some of these polymorphisms may affect the binding sites for transcription factors (HNF-3alpha, C/EBPβ, GR-alpha) and the generation of novel exonic splicing enhancers. Additionally, polymorphism rs55912869 was identified as a potential binding site for the microRNA hsa-mir-3163. Our findings highlight the significant role of *ABCB1* polymorphisms in cognitive decline, particularly in language function, among individuals with amnestic MCI. These polymorphisms may influence gene expression and function through interactions with miRNAs, transcription factors, and alternative splicing mechanisms.

Mild cognitive impairment (MCI), which often progresses to dementia syndrome, varies in its rate of progression among individuals (1,2). A majority of MCI patients, especially those with amnestic MCI (aMCI), progress to Alzheimer’s disease (AD) dementia (2,3). The prevalence of MCI among individuals aged 75 and older ranges from 27% to 44% (4,5). Numerous genetic polymorphisms have been identified that increase the risk of developing AD or are associated with various clinical phenotypes, including the age of onset, rate of progression, and clinical subtype (6,7). Similarly, in MCI, the relationship between genetic polymorphisms and various clinical or neuropsychological characteristics of patients can be studied. Polymorphisms in specific genes, such as *APOE*, cystatin C (*CST3*), cholesterol 24-hydroxylase (*CYP46*) (8), and phosphatidylinositol-binding clathrin assembly protein gene (*PICALM*) (9), may be associated with the risk of development of MCI due to AD or AD itself. In some cases, the impact of polymorphisms on MCI due to AD risk only becomes apparent when they interact with another gene variant, particularly *apoE4* (10).

The *ABCB1* gene (also known as *MDR1* or *PGY1*) encodes the ATP-dependent translocase ABCB1, a glycosylated integral plasma membrane protein (P-glycoprotein 1) and a member of the family of ATP-binding cassette (ABC) transporters. This protein acts as an ATP-dependent enzyme that translocates endobiotics and xenobiotic compounds, including drugs and phospholipids, across the membrane (11). It is also a floppase involved in the flopping of cholesterol, phosphatidylcholine, phosphatidylethanolamine, beta-d-glucosylceramides, and sphingomyelins from the cytoplasmic to the exoplasmic leaflet of the apical membrane. ABCB1 is expressed in the plasma membranes of various cells and organs, including the endothelium of the blood-brain barrier, and functions as a transport pump that moves a variety of compounds from the brain back into the blood (12). ABCB1 has been reported to reside in lipid rafts and to interact closely with cholesterol (13).

ABCB1 plays at least 2 fundamental roles in the pathogenesis of AD. Firstly, ABCB1 actively transports amyloid-beta (Aβ) peptides and a wide range of neurotoxic compounds and xenobiotics from the brain across the blood-brain barrier (BBB) into the blood, reducing their accumulation in the brain; thus, ABCB1 protects the brain from potential damage. The role of ABCB1 in the clearance of Aβ is well-established (14,15). Both Aβ40 and Aβ42 peptide isoforms are substrates for ABCB1 (14). Impaired ABCB1 function leads to the accumulation of Aβ in the brain (16), which, in turn, can decrease the expression of ABCB1 (17). Studies have shown that cholesterol can modulate the activity of secretases involved in Aβ production, and ABCB1 aids in the efflux of Aβ from the brain.

Secondly, ABCB1 also plays a role in lipid transport, including cholesterol, which is essential for neuronal function and integrity (18). ABCB1 activity at the BBB helps maintain cholesterol homeostasis in the brain. Dysregulation of ABCB1 can lead to altered cholesterol levels in the brain, which is a significant factor in the progression of AD (18). Although ABCB1 is predominantly expressed in endothelial cells on the luminal surface of the BBB (15), its presence in pericytes, astrocytes, neurons, and choroid plexus epithelia has also been demonstrated (14,19). Neuro-inflammatory cytokines such as IL-1β, IL-6, and TNF-α can modulate the expression and activity of ABCB1 at the BBB (20).

Aβ and cholesterol are key players in AD pathology, directly affecting neuronal health and cognitive functions. Accumulation of Aβ disrupts synaptic transmission, induces oxidative stress, and triggers neuronal death, particularly in regions critical for memory, such as the hippocampus and prefrontal cortex. Similarly, dysregulated cholesterol levels impair membrane integrity and synaptic plasticity, further exacerbating cognitive decline. Given that ABCB1 influences both Aβ clearance and cholesterol transport, studying its polymorphisms offers crucial insights into the mechanisms underlying early cognitive changes and the progression from MCI to AD (for review, see (21)).

Chen et al. identified ABCB1 as a novel biomarker for AD and MCI (22). Several polymorphisms of the *ABCB1* gene, particularly the C1236T (rs1128503), G2677T/A (rs2032582), and C3435T (rs1045642), have been associated with an increased risk of AD (23,24). Relationships between *ABCB1* polymorphisms and the response to the treatment of patients with AD or other neurological disorders have also been observed (19). However, most studies have focused exclusively on AD patients, omitting those with MCI and lacking data on the association between *ABCB1* polymorphisms and MCI. Frankfort et al. attempted to identify an association between *ABCB1* single nucleotide polymorphisms (SNPs) and various dementing conditions, including the MCI stage. They found no significant relationships in either MCI patients or those with other forms of dementia (25). Searching through available citation databases uncovered no other publications describing the relationship between *ABCB1* SNPs and MCI.

In this study, the objective was to utilize next-generation sequencing (NGS) to analyze the entire *ABCB1* gene in a well-defined longitudinal cohort of cognitively impaired subjects from the Czech Brain Aging Study (CBAS) (26). We examined a broad neuropsychological profile for each participant, complemented by standard demographic parameters. Data were collected from subjects with AD, MCI, subjective cognitive decline (SCD), and cognitively healthy older adults (controls). The inclusion of healthy controls (HCs) in this study was designed to determine whether *ABCB1* gene polymorphisms influence cognitive functions beyond the pathological spectrum of dementia. Healthy controls provide a critical reference to distinguish changes specific to MCI from normal age-related alterations, contributing to our understanding of the role of these polymorphisms in relation to cognitive performance in the early stages of cognitive decline. By analyzing psychological traits and disease characteristics in relation to genetic variants within this well-defined memory clinic-based cohort, we aimed to uncover novel associations between genotypes and phenotypes that may have been overlooked in larger and more heterogeneous studies (27). The primary hypothesis of this study is that polymorphisms in the *ABCB1* gene influence cognitive performance by modulating key processes involved in AD pathology, such as Aβ clearance and cholesterol transport. These processes are critical for neuronal health, synaptic function, and the prevention of neurodegeneration in regions essential for memory and cognition, such as the hippocampus. The aim of this study is to explore associations between *ABCB1* polymorphisms and specific cognitive domains, providing a foundation for future mechanistic research.

## Method

### Participants

The study recruited a total of 1 005 participants from the CBAS cohort at the Memory Center of the International Clinical Research Center at St. Anne’s University Hospital in Brno, and the Memory Clinic of the Department of Neurology at Charles University, Second Faculty of Medicine and Motol University Hospital in Prague, as previously described (26). Participants with AD (*n* = 250), aMCI (*n* = 386), non-amnestic MCI (naMCI) (*n* = 90), and SCD (*n* = 228) were referred by general practitioners, neurologists, and psychiatrists due to subjective cognitive complaints reported by themselves or their close informants. Healthy controls (*n* = 51) were recruited from the University of the Third Age, senior centers (eg, the Elpida Center), or were relatives of participants and hospital staff. All participants underwent standard clinical and laboratory assessments, comprehensive medical history taking, brain MRI, and neuropsychological examinations at the start of the study and then annually, except for HC and SCD participants who were examined biannually. The number of observations per participant ranged from 1 to 5, depending on their year of inclusion in the study. Nondemented participants were followed up until they progressed to dementia, with identical follow-up procedures as during the baseline visit. Participants with dementia syndrome were classified according to the type of dementia using only the baseline data, as they were not followed up.

The participants with AD met the published criteria for probable AD (28) including a gradual onset of symptoms, a clear decline in cognitive function from previous levels, and the absence of any other major psychiatric or neurological disorder that could explain the symptoms. The participants with aMCI and naMCI met the published clinical criteria for MCI (29) including self-reported memory complaints, evidence of cognitive impairment on neuropsychological testing, generally intact activities of daily living, and the absence of dementia. The MCI participants with memory impairment were classified as aMCI, whereas those with impairment limited to nonmemory domains (executive, language, and/or visuospatial functions) were classified as naMCI. Both aMCI and naMCI groups included single- and multiple-domain phenotypes. Cognitive impairment was established if the participant scored more than 1.5 standard deviations (*SD*) below the age- and education-adjusted norms on any test within the cognitive domain. SCD participants met the published criteria for SCD (30) including self-reported cognitive decline within the last 5 years unrelated to an acute event, with cognitive test performance within the normal range adjusted for relevant demographic variables. The HC participants did not report any subjective cognitive complaints requiring medical attention, as confirmed in a structured interview conducted by an experienced neuropsychologist. Their cognitive performance on comprehensive neuropsychological assessment was within the normal range (that is, none of the scores was ≥1.5 *SD* below the age- and education-adjusted norms). Diagnoses were made at joint meetings of neuropsychologists and neurologists, based on mutual agreement.

Individuals below the age of 55, those with a history or evidence of neurological diseases that may affect cognition (such as traumatic brain injury, and neuroinfection), individuals with a history of psychiatric diseases (including major depressive disorder, bipolar affective disorder, generalized anxiety disorder, and schizophrenia), and those with abnormal neurological signs such as gait or movement difficulties were excluded from the study. The detailed criteria for inclusion and exclusion are described elsewhere (26).

All participants in the study provided written informed consent, and the study procedures were approved by the ethics committees of St. Anne’s University Hospital Brno and Motol University Hospital (Prague, Czech Republic) and were conducted in accordance with the Helsinki Declaration of 1975 (revised 2000).

### Neuropsychological Assessment

Following assessment of overall cognitive function using the Mini-Mental State Examination (MMSE), the participants completed several neuropsychological tests to evaluate their performance in 5 cognitive domains. These domains included attention and working memory, memory, executive function, language, and visuospatial function. The attention and working memory domain was measured using the Forward and Backward Digit Span Subtests (DS-F and DS-B), as well as the Trail Making Test (TMT) A. Memory was assessed using the Logical Memory test, delayed recall after 20 minutes, the Rey Auditory Verbal Learning Test (RAVLT) sum of trials 1 to 5 and delayed recall after 30 minutes (RAVLT-DR), and the Enhanced Cued Recall free and cued recall (ECR-FR and ECR-TR). Executive function was evaluated with the TMT B and COWAT (phonemic verbal fluency tests—P-VF, Czech version with letters N, K, P), whereas language was measured using the Boston Naming Test (BNT) 30-odd-items version and the semantic verbal fluency test (S-VF) with animals and vegetables. Visuospatial function was assessed with the Rey-Osterrieth Complex Figure Test (ROCFT), the copy condition, and the Clock Drawing Test (CDT). A composite domain score was calculated for each cognitive domain by averaging the *z*-scores of each test within that specific domain. *Z*-scores were calculated for each test by comparing the participant’s score to the mean and standard deviation of HC participants from the first examination. The *z*-scores for TMT A and B were multiplied by −1 to match the direction of the other neuropsycho‑logical values. The memory domain composite score varied between the SCD and aMCI groups and group with AD, as the RAVLT was only administered to SCD and aMCI patients due to its difficulty for those with dementia. Instead, the ECR was used for patients with dementia. If a participant was unable to complete the TMT A or B in the designated time, they were given a score of 151 seconds or 301 seconds, respectively. Supplementary Table S1 summarizes the neuropsychological tests used to assess cognitive domains.

### Next-Generation Sequencing

The samples for NGS were processed in accordance with the protocol described in Šerý et al. (31). In brief, blood samples were processed using a Prepito NA Body Fluid Kit (Chemagen, PerkinElmer, Waltham, MA), and the DNA concentration was measured using an iQuant dsDNA HS Assay Kit (ABP Biosciences, Beltsville, MD). Library preparation was conducted using the SeqCap EZ System (Roche Sequencing Solutions, Pleasanton, CA), with probes targeting the complete ABCB1 gene, in accordance with the manufacturer’s instructions. The indexed paired-end libraries were prepared using 200 ng of genomic DNA and the KAPA HyperPlus Library Preparation Kit and KAPA Dual-Indexed Adapter Kit (both Roche), according to the manufacturer’s instructions. The quality of the amplified sample libraries was determined using the iQuant dsDNA HS Assay Kit and Fragment Analyzer 5200 (Agilent, Santa Clara, CA). Sixteen amplified DNA sample libraries were combined into a single library pool (with a combined mass of 1 µg) and hybridized with probes targeting 73 genes, including ABCB1, using the HyperCap Target Enrichment Kit, HyperCap Bead Kit, and SeqCap EZ probe pool (all Roche Sequencing Solutions). The quality and concentration of the captured libraries were determined using a Fragment Analyzer 5200, and paired-end 2 × 150 bp sequencing was performed using the Illumina NextSeq 500 Sequencer using NextSeq 500/550 High Output Kit v2.5 (150 Cycles; Illumina, San Diego, CA).

The NGS data were processed and analyzed using the GATK pipelines for variant discovery and germline short variant discovery, in accordance with the recommended procedures for data preprocessing (32). The reference genome used for alignment of reads was Hg38, and the BWA 0.7.13 software package (33) was employed for this purpose. The remaining analyses were conducted using the GATK4 4.1.2.0 software package (34). Only polymorphisms whose genotypes were determined in at least 50 samples with a depth of coverage of greater than 50 reads were subjected to further statistical evaluation.

### Prediction of the Effects of Polymorphisms on miRNA Binding Sites

MicroRNAs (miRNAs) play critical roles in the posttranscriptional regulation of gene expression, either through mRNA cleavage or translational repression (35). SNPs in the miRNA target sites may change the binding efficiency between miRNA and mRNA. We analyzed the potential role of polymorphic sites that were significantly associated with a particular group in the predicted miRNA target sites using the miRBase database (36). We selected sequences 25 bases upstream and downstream of polymorphism sites, limiting the search to sequences of human miRNAs.

### Prediction of the Effects of Polymorphisms on Transcription Factor Binding Sites

Transcription factor binding sites (TFBSs) were analyzed using the software PROMO v3.0.2, which utilizes TRANSFAC v8.3. The potential promoter regions, represented by the *ABCB1* intronic sequences containing individual polymorphisms, were loaded as the query sequence to search for potential binding sites. Predictions were made utilizing only sites that bind human transcription factors, with a dissimilarity threshold parameter set at 10% (90% similarity).

### Prediction of the Effects of Polymorphisms on Splice Sites

To assess the impact of polymorphisms on splicing efficiency, computational analysis was conducted using HSF 3.1 and ESE finder 3.0 bioinformatics software. HSF 3.1 identifies the locations of the receptor (3’ss) and donor (5’ss) splice sites using a position weight matrix approach. ESE finder 3.0 was employed to estimate alterations in ESE sequences as a consequence of mutations. The default threshold setting was used to identify loci responsible for the interaction with 4 serine/arginine-rich (SR) proteins, including ASF/SF2 (alternative splicing factor/splicing factor 2), SRp40, SC35, and SRp55. Only sequences with scores at or above the threshold settings were selected.

### Statistical Analysis

Statistical analysis was conducted separately for each group (ie, HC, SCD, naMCI, aMCI, and AD). The following variables were computed for each participant and used in the statistical analysis: (1) the age at the time of the first examination (AGE); (2) the domain residual *z*-score at the first examination for each domain (ie, AWM_SC, MEM_SC, EF_SC, LG_SC, and VS_SC); and (3) the slope of a simple ordinary least square regression line for the development of the domain residual *z*-score during the 2 years after the first examination with the time (calculated in days) elapsed since the first examination as an independent variable for each domain (ie, AWM_DIF, MEM_DIF, EF_DIF, LG_DIF, and VS_DIF) if repeated examinations were available. The domain residual *z*-score values from the first and 2 subsequent examinations (which were conducted at around 1-year intervals) were utilized to calculate AWM_DIF, MEM_DIF, EF_DIF, LG_DIF, and VS_DIF. Consequently, these variables represent the average increase or decrease in the domain residual *z*-score per year.

*Z*-scores were calculated by using the mean and standard deviation values of the HC group. To remove the effect of age, a regression model was created with AGE as an independent variable, which was calculated based on the HC group values. The residual values from this regression model were calculated for AWM_SC, MEM_SC, EF_SC, LG_SC, and VS_SC. These residual values were used for all subsequent statistical analyses instead of the original *z*-scores. The same process was used for *z*-scores obtained during follow-up examinations of patients, if available. Therefore, AWM_DIF, MEM_DIF, EF_DIF, LG_DIF, and VS_DIF values were also calculated based on residual *z*-scores.

The study investigated the relationship between specific variables and SNPs in the *ABCB1* gene using the Kruskal–Wallis test. To correct for multiple comparisons, a significance level was calculated for each variable by performing an iteration procedure in each group. During each iteration, sequencing data were randomly assigned to patients within the group, and the association of each variable with all single nucleotide polymorphisms (SNPs) was tested. The lowest obtained *p* value for each variable was saved, and after 1 000 iterations, the 5th percentile of minimum *p* values was calculated as the corrected significance level of .05 for each variable. A corrected significance level of .1 was also determined to identify results approaching significance. These levels were used to determine the statistical significance of the association between variables and SNPs. The study also assessed linkage disequilibrium for associated polymorphisms using the *R*^2^ measure, and all statistical calculations were performed using the R software (R Foundation for Statistical Computing, Vienna, Austria).

## Results

Regarding the participants’ demographic characteristics and cognitive assessments of specific cognitive deficits, significant differences were observed among the groups. The study population was recruited from various sources to represent a spectrum of cognitive states. The HC group consisted of individuals who were primarily engaged in educational programs for seniors, such as the University of the Third Age, which reflects their active interest in lifelong learning. In contrast, participants with aMCI and AD were identified through clinical settings, including general practitioners, neurologists, and psychiatrists, based on self-reported cognitive complaints or observations by their families. Differences in age distribution between the groups were inherent to the study design, as aMCI typically occurs earlier than AD. Consequently, the aMCI group was on average younger than the AD group, whereas the HC group was closer in age to the aMCI group to capture the earliest stages of cognitive decline. The recruitment methods and population characteristics contributed to variability in demographic factors, such as education and gender, ensuring a diverse representation of cognitive states. Cognitive performance scores exhibited a gradual decline across the groups, starting from those with normal cognition (ie, HC and SCD), to participants with both types of MCI, and finally to those with AD. Complete descriptive statistics for all groups are presented in Table 1.

**Table 1.** Descriptive Statistics for All Groups of Patients Involved in the Study

		HC		SCD		naMCI		aMCI		AD	*p* Values
	*N*	Mean ± *SD*	*N*	Mean ± *SD*	*N*	Mean ± *SD*	*N*	Mean ± *SD*	*N*	Mean ± *SD*	
Age (years)	51	69.18 ± 7.47	228	66.81 ± 7.86	90	69.26 ± 6.78	386	72.84 ± 7.51	250	74.26 ± 7.88	<.0001
Male	28	7 (25%)	189	62 (33%)	90	24 (27%)	281	128 (46%)	144	66 (46%)	.0008
Female	28	21 (75%)	189	127 (67%)	90	66 (73%)	281	153 (54%)	144	78 (54%)	.0008
Education (years)	26	16.04 ± 1.84	188	15.3 ± 2.95	90	13.58 ± 3.1	188	14.44 ± 3.13	101	13.82 ± 2.91	<.0001
MMSE	45	28.16 ± 3.94	220	28.69 ± 1.66	90	27.53 ± 2.09	378	25.53 ± 3.25	239	20.46 ± 4.73	<.0001
MEM_SC	38	0 ± 0.67	217	−0.25 ± 0.64	87	−0.49 ± 0.77	321	−1.79 ± 0.96	199	−0.89 ± 0.85	—^†^
MEM_DIF	0		119	0.14 ± 0.27	49	0.13 ± 0.3	62	0.12 ± 0.5	1	0.07	—^†^
EF_SC	37	0 ± 0.75	221	−0.19 ± 0.97	90	−1.27 ± 1.21	334	−1.64 ± 1.49	161	−2.68 ± 1.86	<.0001
EF_DIF	0		121	0.1 ± 0.37	49	0.16 ± 0.53	71	−0.08 ± 1.22	1	1.5	.037
LG_SC	36	0 ± 0.48	214	−0.19 ± 0.49	89	−0.65 ± 0.52	330	−0.94 ± 0.67	182	−1.56 ± 0.73	<.0001
LG_DIF	0		120	0.05 ± 0.22	48	0.08 ± 0.23	74	−0.07 ± 0.33	1	0.4	.0105
VS_SC	33	0 ± 0.52	213	−0.19 ± 0.78	89	−0.71 ± 0.92	302	−1.32 ± 1.66	165	−3.25 ± 2.18	<.0001
VS_DIF	0		119	0.93 ± 10.99	49	0.04 ± 0.59	72	−0.28 ± 1.05	1	0.55	.1226
AWM_SC	33	0 ± 0.67	220	−0.12 ± 0.79	89	−1.11 ± 0.74	348	−1.16 ± 0.93	205	−1.86 ± 1.15	<.0001
AWM_DIF	0		121	0.01 ± 0.36	49	0.12 ± 0.49	74	−0.05 ± 0.54	1	0.48	.1806
Body height (cm)	25	166.92 ± 7.34	182	169.3 ± 10.71	80	167.03 ± 7.59	188	168.47 ± 9.61	79	169.48 ± 10.18	.2646
Body weight (kg)	25	74.68 ± 11.81	181	76.1 ± 15.83	80	80 ± 58.88	188	75.26 ± 14.44	79	72.41 ± 13.36	.5499
BMI	25	26.75 ± 3.63	181	27.29 ± 15.64	80	28.76 ± 21.91	188	26.43 ± 4.19	79	25.13 ± 3.67	.1238
Hypertension	33	19 (58%)	169	84 (50%)	66	31 (47%)	217	111 (51%)	105	56 (53%)	.8577
Hypercholesterolemia	31	14 (45%)	159	79 (50%)	57	29 (51%)	207	77 (37%)	101	41 (41%)	.1116
CAD	30	1 (3%)	162	8 (5%)	62	3 (5%)	206	19 (9%)	99	14 (14%)	.0746
Congestive heart failure	31	0 (0%)	164	2 (1%)	63	3 (5%)	213	7 (3%)	91	4 (4%)	.3453
CLTI	30	0 (0%)	149	11 (7%)	56	1 (2%)	203	11 (5%)	92	7 (8%)	.3446
Stroke	29	2 (7%)	158	16 (10%)	62	10 (16%)	203	19 (9%)	95	8 (8%)	.5621
Diabetes	31	1 (3%)	163	15 (9%)	62	9 (15%)	216	28 (13%)	94	23 (24%)	.0063

*Notes:* AD = Alzheimer disease; aMCI = amnestic mild cognitive impairment; AWM_DIF = average increment of AWM_SC per year during the 2 years since the first examination (intercept of the OLS regression line); AWM_SC = attention and working memory domain residual *z*-score at the first examination; BMI = body mass index; CAD = coronary artery disease; CLTI = chronic limb-threatening ischemia; EF_DIF = average increment of EF_SC per year during the 2 years since the first examination (intercept of the OLS regression line); EF_SC = executive functions domain residual *z*-score at the first examination; HC = healthy control; LG_DIF = average increment of LG_SC per year during the 2 years since the first examination (intercept of the OLS regression line); LG_SC = language domain residual *z*-score at the first examination; MEM_DIF = average change of MEM_SC per year during the 2 years since the first examination (intercept of the OLS regression line); MEM_SC = memory domain residual *z*-score at the first examination; MMSE = Mini-Mental State Examination; naMCI = non-amnestic mild cognitive impairment; SCD = subjective cognitive decline; *SD* = standard deviation; VS_DIF = average increment of VS_SC per year during the 2 years since the first examination (intercept of the OLS regression line); VS_SC = visuospatial memory domain residual *z*-score at the first examination.

^†^ *z*-scores are not comparable between groups because different cognitive tests were used (see Supplementary Table S1 for details).

The *p* values presented in the Table 1 are provided for descriptive purposes to highlight statistically significant differences between groups. These values should be interpreted as indicators of potential variation rather than evidence of causal relationships.

### Results of NGS Sequencing

**Figure 1.**
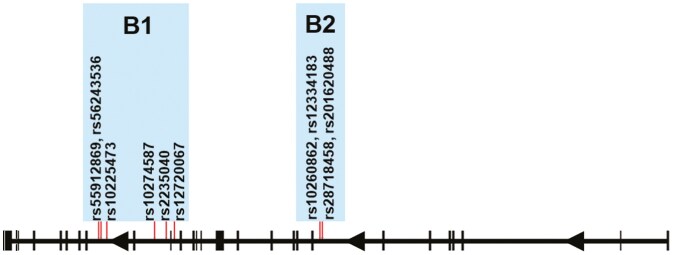
Position of 10 polymorphisms in the *ABCB1* gene. The positions of *ABCB1* polymorphisms significantly associated with the tested variables are marked by red vertical lines.

The entire *ABCB1* gene was sequenced using the NGS method from positions 87503863 to 87713323, following genome assembly GRCh38 (NCBI), resulting in a total of 258 polymorphic sites. After correction for multiple comparisons, 10 polymorphisms were found to be significantly associated with the tested variables (Table 2). Furthermore, these polymorphisms were significantly associated with a greater decline in language performance during the 2-year follow-up period in the aMCI group (*p* < .05 after correction for multiple comparisons). All of these polymorphisms were located in the intronic regions, more than 100 base pairs away from exon-intron boundaries, with the exception of the SNP rs2235040 (Figure 1).

**Table 2.** Association of Selected Polymorphisms of *ABCB1* Gene With Used Variables

SNP	Position/Region	Group	Variable	Genotype 1	Genotype 2	Genotype 3	*p* Value
rs55912869	87523821/intron	aMCI	LG_DIF	T/T −0.13 ± 0.33 (*n* = 54)	T/C 0.21 ± 0.28 (*n* = 12)		.001^*^
rs56243536	87523900/intron	naMCI	MEM_DIF	C/C 0.19 ± 0.27 (*n* = 37)	C/T −0.05 ± 0.38 (*n* = 10)		.0426
		aMCI	LG_DIF	C/C −0.14 ± 0.33 (*n* = 52)	C/T 0.21 ± 0.28 (*n* = 12)		.0011^*^
rs10225473	87525330/intron	AD	EF_SC	A/A −2.57 ± 1.62 (*n* = 63)	A/G −3.91 ± 1.09 (*n* = 13)		.0164
		aMCI	LG_DIF	A/A −0.14 ± 0.34 (*n* = 47)	A/G 0.21 ± 0.28 (*n* = 12)		.0012^*^
		naMCI	VS_DIF	A/A −0.05 ± 0.49 (*n* = 35)	A/G 0.54 ± 0.84 (*n* = 8)		.0425
rs10274587	87535167/intron	AD	EF_SC	G/G −2.59 ± 1.78 (*n* = 89)	G/A −3.79 ± 1.07 (*n* = 14)		.0425
		aMCI	LG_DIF	G/G −0.14 ± 0.33 (*n* = 52)	G/A 0.21 ± 0.28 (*n* = 12)		.0011^*^
rs2235040	87536434/intron	AD	EF_SC	C/C −2.64 ± 1.81 (*n* = 94)	C/T −3.81 ± 1.09 (*n* = 15)	T/T −2.84 ± 1.31 (*n* = 3)	.0439
		aMCI	LG_DIF	C/C −0.14 ± 0.32 (*n* = 55)	C/T 0.21 ± 0.28 (*n* = 12)		.0008^*^
rs12720067	87540040/intron	aMCI	LG_DIF	C/C −0.14 ± 0.32 (*n* = 56)	C/T 0.21 ± 0.28 (*n* = 12)		.0008^*^
rs12334183	87572064/intron	aMCI	LG_DIF	T/T −0.15 ± 0.33 (*n* = 45)	T/C 0.16 ± 0.28 (*n* = 18)		.0008^*^
		aMCI	AWM_SC	T/T −1.1 ± 0.92 (*n* = 154)	T/C −1.34 ± 0.78 (*n* = 54)	C/C 0.63 ± 0.83 (*n* = 4)	.0027
rs10260862	87572166/intron	aMCI	MEM_DIF	G/G −0.02 ± 0.49 (*n* = 35)	G/C 0.3 ± 0.42 (*n* = 13)		.0424
		aMCI	LG_DIF	G/G −0.16 ± 0.33 (*n* = 43)	G/C 0.18 ± 0.29 (*n* = 16)		.0006^*^
rs201620488	87572180/intron	AD	LG_SC	del/del −1.48 ± 0.64 (*n* = 77)	del/ins −1.84 ± 0.69 (*n* = 20)	ins/ins −0.94 ± 0.75 (*n* = 3)	.0421
		aMCI	LG_DIF	del/del −0.16 ± 0.33 (*n* = 42)	del/ins0.17 ± 0.29 (*n* = 17)		.0004^*^
		HC	AWM_SC	del/del 0 ± 0.35 (*n* = 12)	del/ins 0.81 ± 0.67 (*n* = 7)	ins/ins −0.26 ± 0.71 (*n* = 3)	.0239
rs28718458	87572410/intron	aMCI	LG_DIF	G/G −0.14 ± 0.33 (*n* = 43)	G/A 0.16 ± 0.28 (*n* = 18)		.001^*^
		aMCI	VS_SC	G/G −0.99 ± 1.58 (*n* = 135)	G/A −1.79 ± 2.02 (*n* = 55)	A/A −1.41 ± 0.4 (*n* = 4)	.0103
		aMCI	AWM_SC	G/G −1.09 ± 0.93 (*n* = 152)	G/A −1.37 ± 0.75 (*n* = 55)	A/A 0.19 ± 1.44 (*n* = 4)	.0204

*Notes:* Only those polymorphisms whose association with any of the variables was statistically significant at a significance level of .05 after correction for multiple comparisons are reported (marked with an asterisk following the *p* value). For these, the mean values ± standard deviations of the variables for which *p* values were lower than .05 are given. AD = Alzheimer disease; aMCI = amnestic mild cognitive impairment; AWM_SC = the attention and working memory domain residual *z*-score at the first examination; EF_SC = the domain of the executive function residual *z*-score at the first examination; HC = healthy control; LG_DIF = average increment of LG_SC per year during the 2 years since the first examination (intercept of the OLS regression line); LG_SC = the language domain residual *z*-score at the first examination; MEM_DIF = average increment of MEM_SC (the memory domain residual *z*-score at the first examination) per year during the 2 years since the first examination (intercept of the OLS regression line); *n* = sample size; naMCI = non-amnestic mild cognitive impairment; SNP = single nucleotide polymorphism; VS_DIF = average increment of VS_SC per year during the 2 years since the first examination (intercept of the OLS regression line); VS_SC = the visuospatial memory domain residual *z*-score at the first examination.

^*^ *p* values significant at .05 after correction for multiple comparisons, ± *p* values significant at .1 after correction for multiple comparisons.

**Figure 2.**
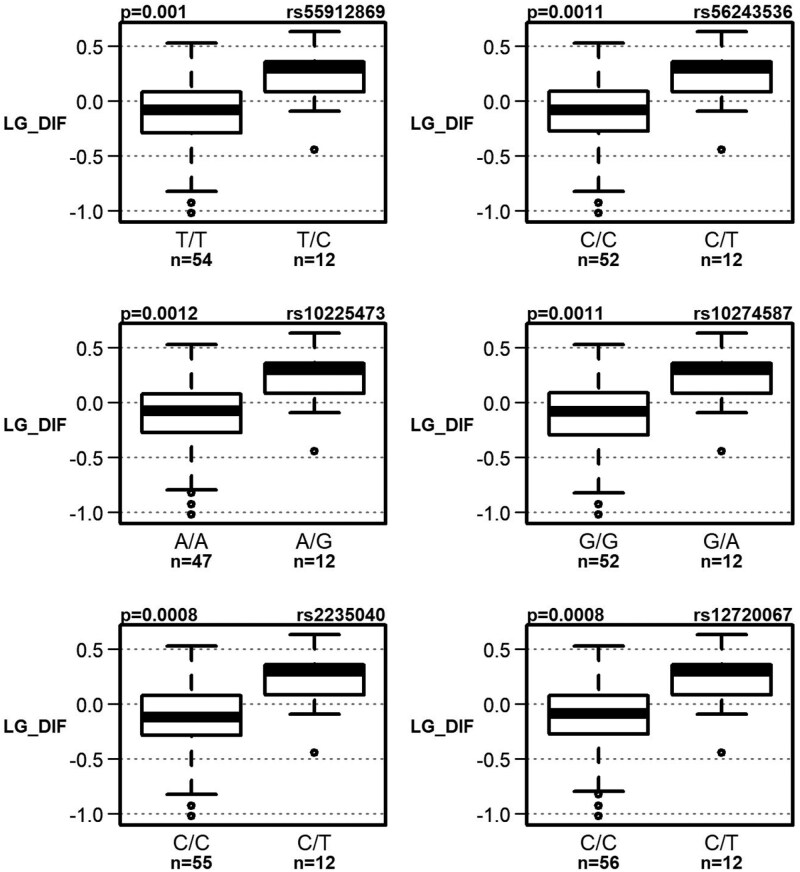
Association of 6 polymorphisms with a decline in language performance in the aMCI group (statistically significant at a significance level of .05 after correction for multiple comparisons).

**Figure 3.**
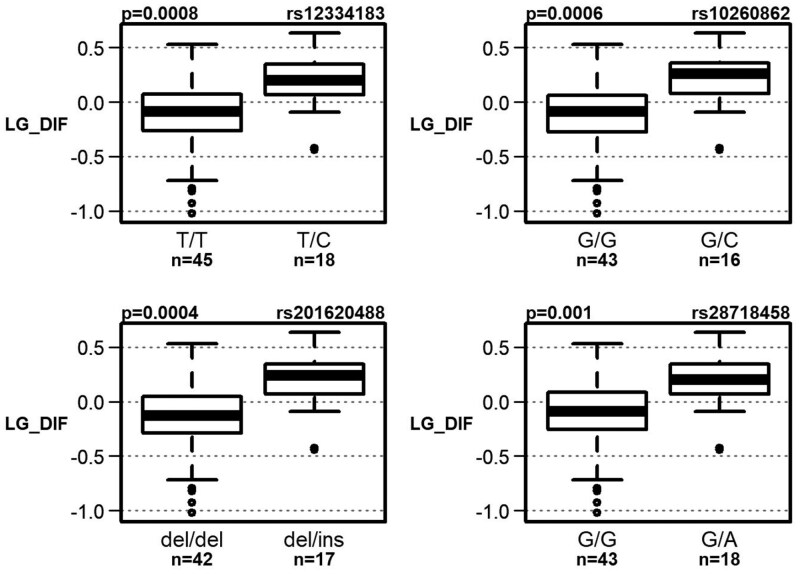
Association of 4 polymorphisms with a decline in language performance in the aMCI group (statistically significant at a significance level of .05 after correction for multiple comparisons).

Interestingly, linkage disequilibrium analysis revealed that these polymorphisms clustered into 2 moderately correlated blocks (Figure 1 and Supplementary Figure S1). Boxplots for each block of polymorphisms are shown in Figures 2 and 3. Detailed results of the observed associations between these 10 polymorphisms and the cognitive test results are presented in Table 2.

### The Effects of Deep Functional Intronic SNPs

Intronic regions located more than 100 base pairs away from exon-intron borders are known as deep intronic regions (37). Although long overlooked, these regions are now recognized as important, and mutations or polymorphisms in these areas have been associated with many diseases. Deep intronic point mutations, deletions, and insertions are considered responsible for these conditions, with the former being the most common (37). Ten of the *ABCB1* intronic polymorphisms associated with the tested variables (Table 2, Figure 1) were analyzed using the MiRbase database to identify potential binding sites for mature human miRNAs in their vicinity. The search revealed no potential binding sites, except in the case of SNP rs55912869, where a potential site for the microRNA hsa-mir-3163 precursor was identified.

Subsequently, the impact of these 10 polymorphisms on TFBSs was evaluated using PROMO v3.0.2 (38). For the polymorphisms clustered into the second block according to LD analysis, the intronic insertion GA/GAGA in the polymorphism rs201620488 could result in the appearance of a new binding site for TF HNF-3alpha (Supplementary Table S2). Allele C in SNP rs12334183 and allele A in SNP rs28718458 would cause the loss of binding sites for transcription factors C/EBPbeta and GR-alpha, respectively (Supplementary Table S2).

Polymorphisms in cis-elements and splice sites can affect gene splicing. The Human Splicing Finder (HSF) web tool (39) was used to predict the effects of 10 *ABCB1* intronic polymorphisms associated with the tested variables. Supplementary Table S3 shows the predictions obtained from HSF. In SNPs rs10260862 and rs12334183, potential activation of a cryptic acceptor site was detected. Additionally, the prediction of new or disrupted ESE sites was obtained for SNPs rs12720067, rs10274587, and rs10225473. Furthermore, the ability of trans-acting SR proteins to alter binding beyond the binding threshold was predicted by the ESE finder software (40) (Supplementary Table S3). Consequently, the in silico analysis demonstrated that some SNPs resulted in the generation of a novel ESE sequence, which may potentially influence the alternative splicing of the *ABCB1* gene.

## Discussion

Our findings are consistent with previous research that underscores the role of ABCB1 in AD pathophysiology through its involvement in Aβ clearance and cholesterol transport (14,18). Chen et al. identified ABCB1 as a biomarker for AD and MCI, highlighting the importance of the gene in these conditions (22). Our study extends this knowledge by demonstrating associations between specific *ABCB1* polymorphisms in deep intronic regions and cognitive decline.

We found a significant association between *ABCB1* polymorphisms and impairment in language function, specifically assessed through tests such as the BNT and the S-VF. The S-VF test evaluates the ability to generate words within a category (eg, animals or vegetables) in a limited time. Performance in S-VF tasks often declines in the early stages of AD, reflecting impairments in semantic memory and executive function. The BNT assesses naming ability by requiring individuals to name depicted objects. Poor performance on the BNT is common in AD patients, indicating significant disruptions in lexical retrieval and semantic processing (41). Additionally, the BNT has been shown to effectively distinguish between stages of cognitive impairment, including MCI and mild dementia, by considering the effects of automatic credits, which may inflate scores in nonaphasic mild dementia patients (42).

As shown in Table 2, the strongest associations were found between *ABCB1* polymorphisms and the average increment of the language domain per year during the 2 years since the first examination in the aMCI group. Language deficits in MCI, such as difficulties with verbal fluency, naming, and syntactic processing, are critical markers of the potential progression to AD. The meta-analysis by Joubert et al. (2020) supports that semantic memory impairments are consistently present in MCI and serve as early indicators of AD (43). Language deficits in AD often manifest early and can serve as significant indicators of cognitive decline. The language domain in AD is characterized by a gradual decline in the ability to generate words and construct sentences, correlating with the deterioration of semantic memory and other cognitive functions. The results of this study suggest that the rs201620488 polymorphism may be weakly associated with cognitive functions, particularly working memory, even in healthy individuals. However, the inclusion of healthy individuals demonstrated that polymorphisms are not generally associated with cognitive functions in the aging population but rather are linked to changes in cognitive performance in MCI and AD populations.

Although long overlooked, deep intronic regions are now recognized as crucial elements of the genome. Mutations or polymorphisms in these regions have been associated with many diseases. In particular, deep intronic point mutations, deletions, and insertions can lead to the activation of cryptic splice sites or disrupt regulatory elements, resulting in the production of aberrant transcripts. These genetic alterations have been implicated in several monogenic disorders and hereditary cancer syndromes, highlighting the importance of comprehensive genomic analysis that includes these noncoding regions to better understand their role in disease pathogenesis (37).

We performed in silico analyses of TFBSs and exonic splicing enhancers (ESEs) of the 10 polymorphisms identified in relation to language function in patients with aMCI. TFBSs are associated with different genomic features when enhancers contain more than 60% of the identified TFBSs and a further 30% of TFBSs are located near transcription start sites or in promoter-proximal regions (44). There are 3 ways in which TFBS variants of functional relevance can regulate gene expression. TFBSs can be involved in specific histone modifications through interactions between transcription factors and chromatin-modifying enzymes (45), in altering the local DNA methylation profile by modulating DNA methylation (46), or in driving topological genome reorganization and changing the structure of enhancer-promoter loops that contribute to gene regulation (46).

Analysis of TFBSs revealed associations with 3 polymorphisms. In our study, we identified a significant polymorphism, rs12334183, affecting a binding site for C/EBPβ, a member of the CCAAT/enhancer-binding protein family, which has been extensively studied in the context of AD due to its critical role in neuroinflammation and neurodegeneration (47). Elevated levels of C/EBPβ have been observed in the brains of AD patients, particularly in the hippocampus, cortex, and cerebellum, where it upregulates the expression of various pro-inflammatory cytokines and other molecules involved in the inflammatory response within the central nervous system. This neuroinflammation is a key contributor to the progression of AD, leading to synaptic dysfunction, neuronal death, and ultimately cognitive decline (48). We also found that the A allele of SNP rs28718458 results in the loss of binding sites for the glucocorticoid receptor alpha (GRα). GRα, which is activated by cortisol, plays a crucial role in the brain’s stress response, inflammation regulation, and neuronal health (49). Dysregulation of GRα, particularly due to chronic stress and elevated cortisol levels, has been implicated in the progression of AD through mechanisms such as increased Aβ production and tau phosphorylation (50). The association of *ABCB1* polymorphisms with language impairments in MCI might be influenced by the role of GRα in these processes, highlighting the importance of genetic factors in the cognitive decline observed in neurodegenerative diseases. Finally, the polymorphism rs201620488 may create a new binding site for the transcription factor HNF-3alpha, also known as FoxA1 (Forkhead Box A1), which is involved in neurodevelopmental processes (51). Its potential role in relation to AD remains to be explored.

Exonic splicing enhancers are short nucleotide sequences within exons that are crucial for pre-mRNA splicing. They function as binding sites for SR proteins, which are essential for the inclusion of the exon in the final mRNA transcript. Disruptions in ESE sequences due to genetic polymorphisms can lead to improper splicing, resulting in the production of abnormal proteins (40). In our study, the HSF tool was used to predict the effects of *ABCB1* intronic polymorphisms on splicing. The analysis showed that the SNPs rs10225473, rs10274587, rs12720067, rs12334183, and rs10260862 resulted in the generation of novel ESE sequences, which may potentially influence the alternative splicing of the *ABCB1* gene. This may indicate the functional relevance of these polymorphisms in the pathogenesis of MCI and AD.

### Limitations

Our study has a few limitations that should be acknowledged. Based on a common effect size (Cohen’s *d*) of 0.5, which is typically considered a medium effect size in the behavioral sciences, we conducted a power analysis using the software GPower. To achieve a power of 0.8 (80%) with an alpha level of 0.05 (5%), the required sample size for each group in a 2-sided *t* test is approximately 63 participants. Our study included a total of 1 005 participants, distributed across 5 groups: AD (*n* = 250), aMCI (*n* = 386), naMCI (*n* = 90), and SCD (*n* = 228). Each group exceeds the required sample size, ensuring that our study is adequately powered to detect medium-sized effects.

Second, while the *p* values in our study were significant at the traditional threshold of *p* < .05 after correction for multiple comparisons, they were actually much lower, ranging from .001 to .0004. This aligns with the more stringent threshold of *p* < .005 suggested by recent evidence to reduce the likelihood of false positives. This robustness in our statistical findings adds confidence to the validity of our results. Moreover, the uniform association we observed between polymorphisms and the language domain across multiple comparisons strongly suggests a genuine relationship, rather than a random occurrence. If the associations had been randomly distributed across different polymorphisms and cognitive domains, it would have indicated a less reliable hypothesis. Therefore, the consistency and significance of our results support the validity of our findings, though they should still be interpreted with caution and ideally validated in further studies with larger sample sizes.

Replication of findings is a cornerstone of scientific research. Although our study presents novel associations between *ABCB1* polymorphisms and cognitive decline in MCI, we were unable to include a replication cohort within this study due to resource constraints. Future research should aim to replicate these findings in independent cohorts to confirm the robustness and generalizability of our results.

Lastly, our study focused on a specific population from the CBAS, which may limit the generalizability of the findings to other populations with different genetic backgrounds and environmental exposures. Additional studies in diverse populations are necessary to determine the broader applicability of our findings.

## Conclusion

Our study examined the impact of *ABCB1* gene polymorphisms on cognitive decline in a cohort of individuals with MCI, AD, SCD, and cognitively healthy older adults. Utilizing NGS, we identified 10 polymorphisms (rs55912869, rs56243536, rs10225473, rs10274587, rs2235040, rs12720067, rs12334183, rs10260862, rs201620488, and rs28718458) significantly associated with cognitive performance, particularly in language decline in aMCI patients. Among these, polymorphisms rs201620488, rs12334183, and rs28718458 may affect the creation or loss of binding sites for transcription factors HNF-3alpha, C/EBPβ, and GR-alpha. Additionally, polymorphisms rs10225473, rs10274587, rs12720067, rs12334183, and rs10260862 may influence the generation of novel ESE sequences, potentially affecting the alternative splicing of the *ABCB1* gene. These polymorphisms could influence *ABCB1* gene expression and function by potentially affecting transcription factor interactions and alternative splicing, suggesting their role in the cognitive decline observed in neurodegenerative diseases.

In conclusion, this study identified significant associations between *ABCB1* polymorphisms and cognitive performance, particularly in individuals with MCI and AD. These findings suggest that genetic variations in *ABCB1* may contribute to cognitive decline through mechanisms related to Aβ clearance and cholesterol transport. Although the study is exploratory and correlative in nature, it provides a valuable foundation for future research aimed at confirming these associations and uncovering their underlying mechanisms. Larger cohorts and diverse populations, as well as mechanistic studies, will be critical to validate and expand upon these findings and to fully elucidate the biological pathways through which these polymorphisms contribute to cognitive decline.

## Supplementary Material

glaf055_suppl_Supplementary_Tables_S1-S3_Figure_S1

## Data Availability

The data sets generated during and/or analyzed during the current study are available from the corresponding author on reasonable request.
