# Supplementary material for: ABCB1 Gene Polymorphisms and Their Contribution to Cognitive Decline in Mild Cognitive Impairment: A Next-Generation Sequencing Study
Source: J Gerontol A Biol Sci Med Sci. 2025 Apr 1;80(6):glaf055. doi: 10.1093/gerona/glaf055 (PMC12093306; doi:10.1093/gerona/glaf055)
Supplement: glaf055_suppl_Supplementary_Tables_S1-S3_Figure_S1 [file glaf055_suppl_supplementary_tables_s1-s3_figure_s1.zip › Supple Figure and Table/Supplemental material.pdf]

Supplementary material to the manuscript of Šerý et al. *ABCB1* Gene Polymorphisms and Their Contribution to Cognitive Decline in Mild Cognitive Impairment: A Next-Generation Sequencing Study

Number of Suppl. Tables: 3

eTable 1 Summary of neuropsychological tests according to cognitive domains

eTable 2 Sequence variants in the *ABCB1* gene

eTable 3 Predictions about splice site SNPs by HSF and ESE-finder software

Number of Suppl. Figures: 1

eFigure 1 Linkage disequilibrium (R2) for identified polymorphisms in the *ABCB1* gene

e-Table1 Summary of neuropsychological tests according to cognitive domains

Domain	Abbreviation	Neuropsychological Tests
1. Attention and working memory	AWM	1.1 Digit Span Forward (DS-F)<br>1.2 Digit Span Backward (DS-B)<br>1.3 Trail Making Test (TMT) A
2. Memory	MEM	2.1 Logical Memory I, delayed recall after 20 minutes (LM-DR)<br>2.2 Rey Auditory Verbal Learning Test, sum of trial 1-5 (RAVLT 1-5)[a]<br>2.3 RAVLT, delayed recall after 30 minutes (RAVLT-DR)[a]<br>2.4 Enhanced Cued Recall, free recall (ECR-FR)[b]<br>2.5 Enhanced Cued Recall, total recall (ECR-TR)[b]
3. Executive function	EF	3.1 TMT B<br>3.2 COWAT - Phonemic Verbal Fluency (P-VF), Czech version with letters N, K, P
4. Language	LG	4.1 Semantic Verbal Fluency (SV-F), Animals<br>4.2 S-VF, Vegetables<br>4.3 Boston Naming Test (BNT), 30 odd-items version
5. Visuospatial function	VS	5.1 Rey-Osterrieth Complex Figure Test (ROCFT), copy condition

[a] Tests that were administered in subjective cognitive decline (SCD) and amnestic mild cognitive impairment (aMCI) groups only, [b] tests that were administered in group of patients with Alzheimer's disease (AD) only.

eTable 2 Sequence variants in the *ABCB1* gene

Variation and polymorphism	TFBS alterations[a] (Dissimilarity/RE equally)	Association of polymorphisms with LG-DIF
rs201620488 (insGA/GAGA)	+ HNF-3alpha [T02512] (3.50/0.007)	YES
rs12334183 (C)	-C/EBPbeta [T00581] (0.00/0.39)	YES
rs28718458 (A)	GR-alpha [T00337] (8.28/0.20)	YES

Note: LG_DIF = average increment of LG_SC (language domain residual z-score at the first examination) per year during the two years since the first examination; TFBS = Transcription factor binding sites.

[a] PROMO analysis using annotations in TRANSFAC version 8.3 entries

eTable 3 Predictions about splice site single nucleotide polymorphisms by HSF and ESE-finder software

rsIDs	Alleles	Predicted Signal[a]	Interpretation[a]	SR Protein type[b]	Site[b]	Score[b]
rs10225473	A>C	New ESS Site	Creation of an intronic ESE site	SRp40	TCAAATG	2.73
rs10274587	G>A	New ESS Site	Creation of an intronic ESE site	SRp40	CTTCAGC	3.76
		ESE Site Broken	Alteration of an intronic ESS site	SRp55	TTCGGC	3.01
rs12720067	C>T	ESE Site Broken	Alteration of an intronic ESS site	SC35	ATTCCCCA	2.38
rs12334183	T>C	New Acceptor splice site	Potential alteration of splicing	SF2/ASF	CAGCTGT	2.47
rs10260862	G>C	Acceptor splice site	Potential alteration of splicing	SRp55	TATGTA	3.61

[a] HSF (Human Splicing Finder) analysis; [b] ESE exonic splicing enhancer finder software
