# Supplementary figures and images for: ABCB1 Gene Polymorphisms and Their Contribution to Cognitive Decline in Mild Cognitive Impairment: A Next-Generation Sequencing Study
Source: J Gerontol A Biol Sci Med Sci. 2025 Apr 1;80(6):glaf055. doi: 10.1093/gerona/glaf055 (PMC12093306; doi:10.1093/gerona/glaf055)

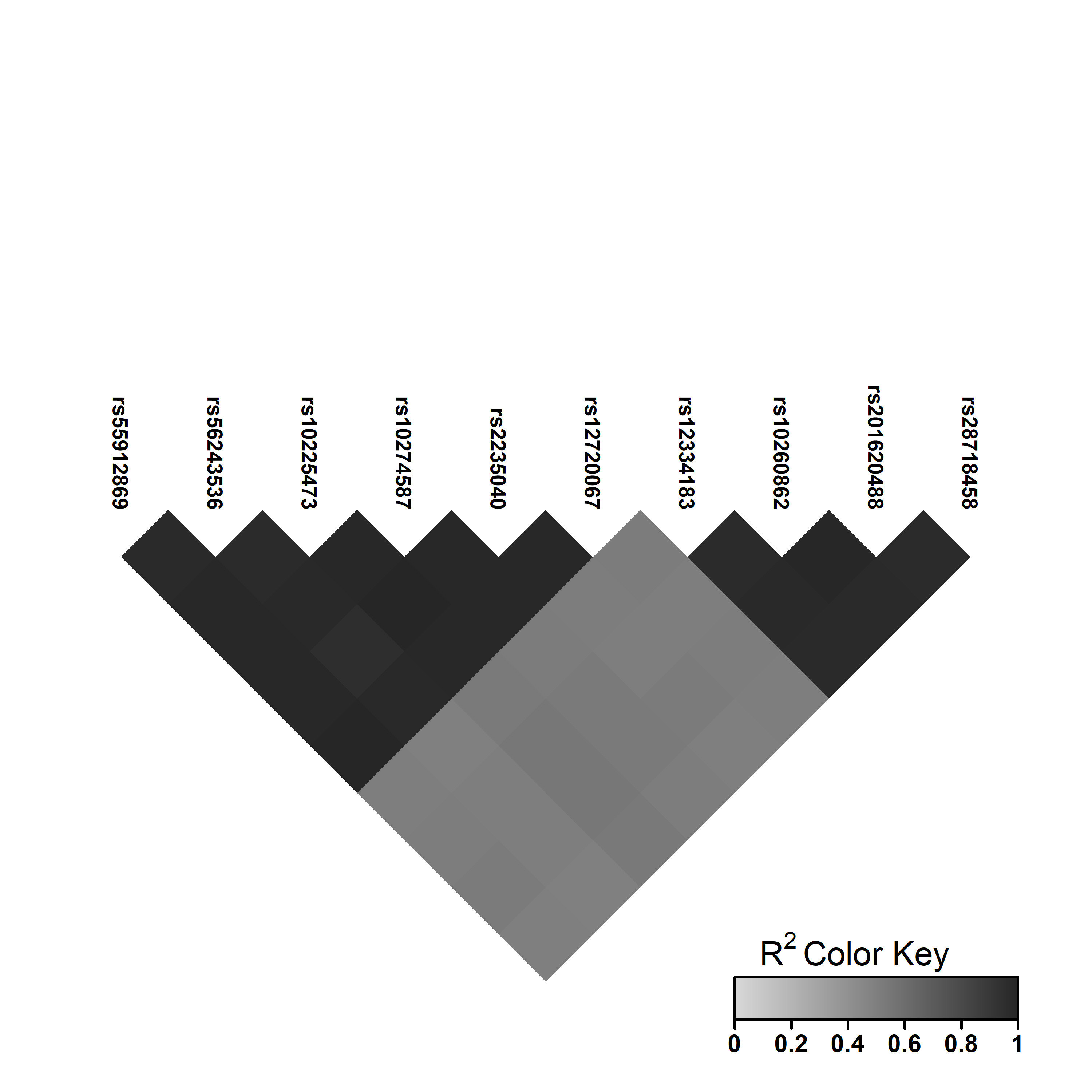

Supplement: glaf055_suppl_Supplementary_Tables_S1-S3_Figure_S1 [file glaf055_suppl_supplementary_tables_s1-s3_figure_s1.zip › Supple Figure and Table/eFigure_1.jpg]
